# Multi-planar instability, laxity and reduced knee flexion during the support phase of walking are determinants of return to sports

**DOI:** 10.3389/fbioe.2022.1047135

**Published:** 2022-11-03

**Authors:** Tianping Zhou, Yihong Xu, Lan Zhou, Siya Wang, Shaobai Wang, Weidong Xu

**Affiliations:** ^1^ Department of Joint Surgery and Sports Medicine, Changhai Hospital Affiliated to Navy Medical University, Shanghai, China; ^2^ Key Laboratory of Exercise and Health Sciences of Ministry of Education, Shanghai University of Sport, Shanghai, China

**Keywords:** anterior cruciate ligament, return to sports, *in vivo*, knee, kinematics

## Abstract

**Background:** After anterior cruciate ligament reconstruction, some patients are not recommended to return to high-level physical activity because they fail to pass return-to-sports tests. The kinematic difference between these patients and those who pass the return-to-sports tests is unclear.

**Methods:** Eighty-two patients who received anatomic single-bundle anterior cruciate ligament (ACL) reconstruction for unilateral ACL injury underwent return-to-sport tests during a hospital visit at a minimum of 9 months (9–11 months) of follow-up. Fifteen patients who passed the return-to-sports tests (RTS group) and fifteen patients who did not (NRTS group) were randomly selected to perform a treadmill walk under dual-fluoroscopic imaging system surveillance for a 6 degrees of freedom kinematic evaluation.

**Results:** Of the 82 patients, 53 passed the return-to-sports tests 9 months after surgery, with a return-to-sports rate of 64.6%. In the stance phase, the NRTS group had a larger anterior tibial translation (1.00 ± 0.03 mm vs. 0.76 ± 0.03 mm, *p* = 0.001), a larger lateral tibial movement (1.61 ± 0.05 mm vs. 0.77 ± 0.05 mm, *p* < 0.001), a larger distal tibial displacement (−3.09 ± 0.05 mm vs. −2.69 ± 0.05 mm, *p* < 0.001), a smaller knee flexion angle (6.72 ± 0.07° vs. 8.34 ± 0.07°, *p* < 0.001), a larger varus angle (−0.40 ± 0.03°VS. -0.01 ± 0.03°, *p* < 0.001) and a larger external rotation angle (1.80 ± 0.05° vs. 1.77 ± 0.05°, *p* < 0.001) than the RTS group. The maximum anterior tibial translation of the NRTS group is also larger than that of the RTS group (3.64 ± 0.42 mm vs. 3.03 ± 0.59 mm, *p* = 0.003).

**Conclusion:** Compared with patients passing RTS tests, those who fail to pass show significant anterior, lateral, and rotational instability; knee laxity; and reduced flexion angle of the knee in the support phase during walking, which may be the possible factors hindering a return to sports.

## Introduction

Anterior cruciate ligament (ACL) reconstruction is the main treatment for an anterior cruciate ligament injury to restore knee stability and, eventual, a return to sports (Kohn et al., 2020). Return to sports (RTS) is often defined as a return to the same level, intensity, and frequency of exercise as those before the injury (Meredith et al., 2020). Whether patients are suitable for returning to sports is usually determined by their performance on the RTS tests. However, under the current RTS criteria, the recent return-to-sports rate in most studies are unsatisfactory, ranging from 15.5% to 64% (Lefevre et al., 2017; Rahardja et al., 2021; Webster et al., 2022). In addition, with the lack of adequate kinematic studies, there is a considerable debate about the value of RTS tests in identifying patients who are at higher risk of reinjury (Gokeler et al., 2022; Hurley et al., 2022). Therefore, studying the kinematic differences between patients who pass the RTS tests and those who fail at 9 months after ACL reconstruction will be of great significance for us to deeply understand the kinematic mechanism of return to sports after ACL reconstruction and the value of RTS tests. The hypothesis in this study was that, compared with the RTS group, the NRTS group would show significant differences in knee kinematics during walking 9 months after ACL reconstruction.

The purpose of the present study was to compare the kinematic differences of the knee joint in the support phase during the level walking process between a return-to-sports (RTS) group and a nonreturn-to-sports (NRTS) group 9 months after ACL reconstruction with the high-speed dual-plane fluoroscopic imaging system (DFIS). The DFIS is a new motion-capture system that can accurately measure the spatial position of the knee joint during movement based on the projections of the bone structure of the knee joint in two different directions under X-ray. The system has been verified by the previous literature and is considered an accurate kinematics measurement equipment with displacement and angle measurement errors do not exceed 0.1° and 0.1 mm (Li et al., 2004; Zhu and Li, 2012).

## Methods

### Study design

**FIGURE 1:**
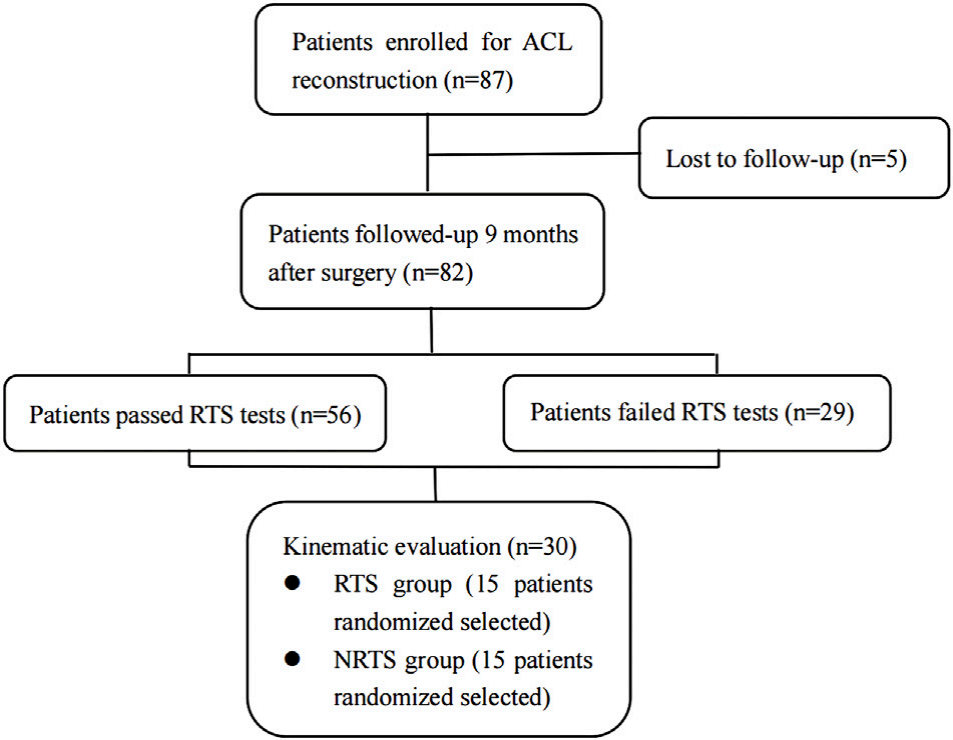
CONSORT (Consolidated Standards of Reporting Trials) flow diagram for kinematic evaluation. ACL, anterior cruciate ligament; ACLR, anterior cruciate ligament reconstruction; RTS, return-to-sports; NRTS, non-return-to-sports.

This study is an observational follow-up study that was approved by the ethics committee of the institution (CHEC 2020097). All patients signed written informed consent forms when they were included in the study. From May 2020 to March 2021, a total of 87 patients who were diagnosed with primary anterior cruciate ligament rupture at Changhai hospital and underwent arthroscopic autologous hamstring tendon single-bundle anterior cruciate ligament reconstruction were included in this study (Figure 1). The purpose of ACL reconstruction was to restore ACL biomechanical function and knee stability which may be the determinants of a return to sports. Based on the parameters of the DFIS and relevant researches, the cumulative radiation dose received by a patient in this study was about 5 mGy, which not exceeded the dose of a typical chest CT scan and shall be considered as safe (Biswas et al., 2009; Xiao et al., 2021).

### Inclusion and exclusion criteria

The inclusion and exclusion criteria were as follows: 1) adult (over 18 years old) male or female patients; 2) diagnosis of ACL rupture under arthroscopy; 3) anatomical single-bundle reconstruction of the ACL with hamstring tendon; and 4) willingness to complete 1 year of regular follow-up. The exclusion criteria were as follows: 1) other severe knee injuries (i.e., collateral ligament, posterior cruciate ligament); 2) ACL reconstruction with other graft types (bone-patellar-tendon-bone, artificial ligament, *etc.*) and surgical techniques; 3) history of severe lower extremity trauma that may affect knee motion (posterior cruciate ligament injury, collateral ligaments injuries, *etc.*); and unwillness to participate in the trial or to complete the follow-up.

### Surgery technique and postoperative management

In this study, the anterior cruciate ligament injury was first determined under arthroscopy and the combined meniscus injury was treated with meniscus suture technique. Then the hamstring tendon was harvested and braided into six strands. With the anteromedial approach, the tibial and femoral tunnels were drilled at the ACL tibial and femoral footprints respectively. The remnant tissue of both sides was preserved in the operation. The femoral and tibial ends of the graft were fixed with Endobutton and Intrafix screws respectively. Finally, range of motion of the knee joint, graft tension and graft impingement were checked under arthroscopy. ACL reconstruction of all patients were performed by the same senior doctor (Dr. Xu).

All the patients were treated with the standard rehabilitation mode as reported (Badawy et al., 2022). During the first 2 weeks after the operation, patients were asked to perform exercises including straight leg raises, ankle pumps, passive/active knee flexion, extension, and hip adduction to control the inflammatory response (swelling, pain, *etc.*); restore the partial range of motion of the knee joint (full extension–90° flexion); restore the patella range of motion; and improve the quadriceps femoris. During 3–5 weeks after the operation, rehabilitation measures such as moving up and downstairs and standing on one leg were used to restore the normal range of motion and normal gait of the knee joint. Patients were allowed for running and jumping 3 months after operation while noncontact sports at 6 months. All the patients were followed up at 2 weeks, 1 month, 3 months, and 6 months after the operation and were given rehabilitation guidance. Return-to-sports and knee kinematics assessments were performed 9 months postoperatively.

### Return-to-sports criteria and tests

According to the Panther consensus in 2020, return-to-sports criteria should include the postoperative time, a subjective score, an objective assessment of knee function, and a psychological readiness assessment (Meredith et al., 2020). On this basis, this study referred to related research and determined the following RTS criteria (Webster and Hewett, 2019). Patients who meet all of the following criteria are considered to pass the RTS tests.

(1) Postoperative time ≥9 months;
(2) Limb symmetry index (LSI) > 90% in three hop tests (single; triple; triple crossover);
(3) IKDC2000 subjective scale >90 points (Rossi et al., 2002);
(4) Anterior cruciate ligament return-to-sports after injury scale (ACL-RSI)>56 points (Webster et al., 2008).

All patients were asked to complete the IKDC2000 and ACL-RSI questionnaires according to their actual situation at the 9-month postoperative outpatient follow-up. All patients were tested with Lachman’s test and a pivot-shift test by senior professional physicians as previously reported (Sokal et al., 2022). The patients were fully informed of the relevant rules and requirements when conducting the hop test. Subjects decide whether to participate in this test according to their knee joint function recovery. If the subject gave up or dared not take the test because of poor knee recovery, the test result was considered unqualified (LSI <90%). The hop tests were performed as described by Barber and Noyes et al. (Barber et al., 1990; Noyes et al., 1991).

### Kinematic evaluation

**FIGURE 2:**
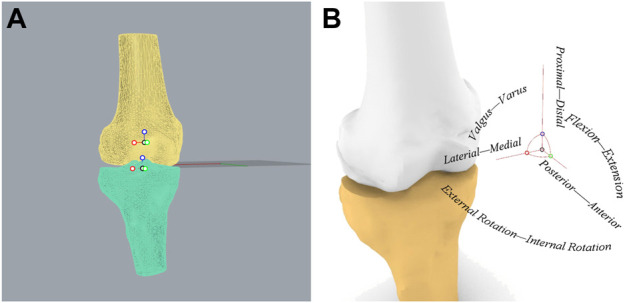
Establishment of knee joint coordinate system. **(A)** Coordinate systems established in the femur and tibia. **(B)** The relationship between the knee joint coordinate system and the kinematics of six degrees of freedom.

Based on the RTS test results, 15 patients from both those who passed the RTS tests (RTS group) and those who failed (NRTS group) were randomly selected for further kinematic examination. CT (Siemens, Germany) images (slice thickness 0.6 mm; resolution 512 × 512pixels) of the operated knee of all patients were acquired. The CT images were imported into 3D modelling software (Amira 6.7; Thermo Fisher Scientific), and the 3D model of the knee joint and the corresponding coordinate systems were established using methods described in previous studies (Rao et al., 2020; Rao et al., 2021). In order to establish the tibial coordinate system, two circles on the tibial plateau plane which were tangent to the edges of the medial tibial plateau and the lateral tibial plateau were found respectively. The straight line through the center of both circles was set as the *X*-axis [medial (−) and lateral (+) axis], and the straight line parallel to the long axis of the tibia through the origin of the coordinate system was set as the *Z*-axis [proximal (+) and distal (−) axis]. A plane was established by the *X*-axis and the *Z*-axis, and the straight line perpendicular to the plane through the origin of the coordinate system was set as the *Y*-axis [anterior (+) and posterior (−) axis]. Then the femoral coordinate system was established, and the transepicondylar axis (TEA) which connected the most prominent points of the medial and lateral epicondyles was set as the *X*-axis [medial (−) and lateral (+) axis]. The midpoint of the TEA was set as the coordinate origin of the femoral coordinate system, and the line passing through this point and parallel to the long axis of the femur was set as the *Z*-axis [proximal (+) and distal (−) axis]. A plane was determined based on the *X*-axis and *Z*-axis, and the straight line passing through the origin of the femur coordinate and perpendicular to the plane was set as the *Y*-axis [anterior (+) and posterior (−) axis] (Figure 2).

In this study, a high-speed dual-plane fluoroscopy system (DFIS) (Shanghai Yidong Medical Technology Co., Ltd., Shanghai, China) was used to collect kinematic images of the knee joint. The preliminary positions of the X-ray transmitter and the receiver were adjusted according to the position of the treadmill to ensure that the images during the entire exercise process could be presented on the receiver from two different directions. The specific operation and use of DFIS were as described in previous studies (Li et al., 2004; Rao et al., 2020). Finally, the X-ray transmitter was adjusted to 60kV and 10 mA to achieve the best shooting effect. The DFIS was adjusted to continuous pulse mode, the exposure time was adjusted to 1/1000 s, and the sampling frequency was adjusted to 200 frames/second.

**FIGURE 3:**
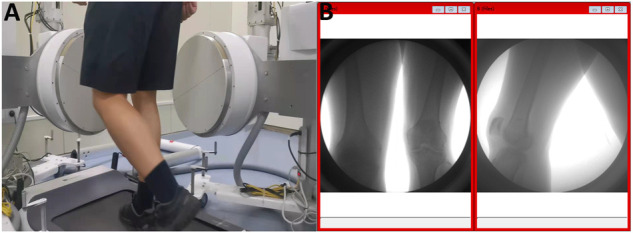
Acquisition of kinematic images of the knee joint. **(A)** Walking on a treadmill at 1 m/s. **(B)** Kinematic images of the knee joint acquired simultaneously in both directions.

A suitable plantar pressure-sensing insole was placed on the subject’s sole pad. Subjects were asked to perform 3 min of adaptive activity on a treadmill before initiation of the sampling. When the sampling officially started, the patients put on a protective lead vest and a protective lead scarf for radiation protection. The patients were asked to stand on the treadmill, and the treadmill speed was slowly adjusted to 1 m/s. After the patients adapted, the images of knee joint motion during a complete gait cycle during flat walking were collected according to the pressure changes of the sole pressure insole (Figure 3).

**FIGURE 4:**
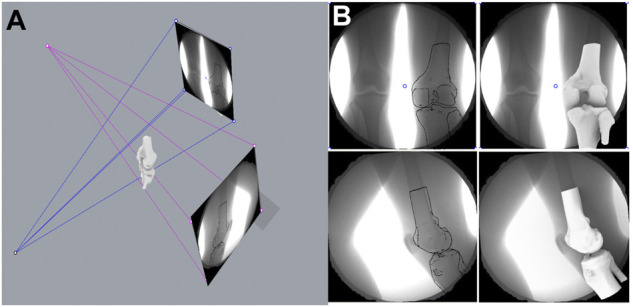
Kinematic measurement of six degrees of freedom of the knee joint. **(A)** Mechanism of the dual-plane fluoroscopic imaging system for measuring knee kinematic indicators. **(B)** Registration of 3-dimension model of knee joint with kinematic images.

Among all the images of the support phase of the gait cycle, the biplane images of the corresponding time points were selected in turn at intervals of 10% of the total time of the support phase. After the selected images and the 3D model of the femur and tibia were imported into MATLAB (R2018a; MathWorks), the spatial position of the femur and tibia model were adjusted to fit the outlines in images from both directions. Finally, the relative positional relationship between the tibia and the femur was calculated (the displacement and rotation angle on the *X*, *Y*, and *Z* axes) (Figure 4).

### Statistical analysis

SPSS 22.0 (IBM SPSS Statistics, United States) software was used for statistical analysis in this experiment. The count data conforming to the normal distribution were expressed as the mean ± standard deviation, and the measurement data were expressed as frequency. For measurement data such as age, height, BMI, and time from surgery, two independent samples t tests or Mann-Whitney U tests were selected according to their normality and homogeneity of variance. Count data were tested by the chi-square test or Fisher’s exact test Table 1. The repeated measures analysis of variance statistical method was used to compare the kinematic indices of the six DOF kinematics of the support phase period of the knee joint between the RTS group and the NRTS group. A *p*-value of <0.05 was considered to be significant. A post hoc power analysis indicated that 15 subjects would provide over 95% power to detect the kinematics changes (shown in Table 2).

**TABLE 1:** Patients and clinical characteristics.

	NRTS group (n = 15)	RTS group (n = 15)	*p*-value
Age (yrs)	27.60 ± 6.05	27.47 ± 6.06	0.952
Mass (kg)	175.60 ± 3.31	175.73 ± 4.32	0.925
Body height (cm)	74.27 ± 8.65	73.53 ± 6.52	0.795
Body mass index (kg/m^2^)	24.09 ± 2.75	23.81 ± 1.91	0.743
Time from surgery (mo)	9.50 ± 0.37	9.43 ± 0.52	0.471
Male/Female	11/4	13/2	0.651
Right/Left	10/5	11/4	0.996
Meniscus injury (Yes/No)	12/3	11/4	0.951
Lachman test			0.475
Normal	12	14	
Grade 1+	2	1	
Grade 2+	1	0	
Grade 3+	0	0	
Pivot-shift test grade			0.505
Normal	11	13	
Grade 1+	3	2	
Grade 2+	1	0	
Grade 3+	0	0	

**TABLE 2:** Comparison of knee kinematics between those who passed the return-to-sports test and those who failed 9 months after ACL reconstruction.^a^ The maximum valgus angle was at 100% stance phase in the NRTS group while 0% in the RTS group. NRTS, not return-to-sports; RTS, return-to-sports; CI, confidence interval; SD, standard deviation; AVG, average value; Max, maximum value; Min, minimum value; A-P, anterior-posterior; L-M, lateral-medial; P-D, proximal-distal; F-E, flexion-extension; Valg-Var, valgus-varus; Er-Ir, external rotation-internal rotation.

		Stance phase (%)	NRTS group				RTS group				*p*-value
			95%CI				95%CI				
			Mean	SD	Lower	Upper	Mean	SD	Lower	Upper	
A-P (mm)	AVG	\	1.00	0.03	0.93	1.07	0.76	0.03	0.70	0.83	<0.001
	Max	30	3.64	0.42	3.37	3.91	3.03	0.59	2.71	3.34	0.003
	Min	0	−2.79	0.59	−3.09	−2.48	−2.21	0.58	−2.52	−1.90	0.01
L-M(mm)	AVG	\	1.61	0.05	1.51	1.71	0.77	0.05	0.67	0.87	<0.001
	Max	100	3.05	0.71	2.68	3.42	2.96	0.69	2.59	3.33	0.729
	Min	80	−0.59	0.42	−0.81	−0.36	−1.94	0.43	−2.17	−1.72	<0.001
P-D (mm)	AVG	\	−3.09	0.05	−3.18	−2.99	−2.69	0.05	−2.79	−2.59	<0.001
	Max	50	−1.74	0.37	−1.93	−1.54	−1.70	0.37	−1.89	−1.50	0.763
	Min	0	−6.34	0.77	−6.73	−5.94	−4.37	0.73	−4.76	−3.97	<0.001
F-E (°)	AVG	\	6.72	0.07	6.57	6.86	8.34	0.07	8.20	8.49	<0.001
	Max	100	21.39	1.54	20.56	22.21	23.74	1.56	22.92	24.56	<0.001
	Min	80	0.18	0.08	0.13	0.22	0.13	0.08	0.08	0.17	0.084
Valg-Var (°)	AVG	\	−0.40	0.03	−0.45	−0.34	−0.01	0.03	−0.06	0.05	<0.001
	Max	100/0	0.68	0.37	0.49	0.87	0.84	0.33	0.66	1.01	0.656
	Min	70	−2.36	0.65	−2.67	−2.04	−2.11	0.54	−2.42	−1.79	0.262
Er-Ir (°)	AVG	\	1.80	0.05	1.69	1.91	1.77	0.05	1.66	1.88	<0.001
	Max	100	7.52	1.05	6.98	8.05	7.46	0.99	6.92	7.99	0.871
	Min	0	−2.77	0.57	−3.06	−2.47	−2.13	0.55	−2.42	−1.83	0.004

## Results

### Patient and clinical characteristics

Of the 87 patients initially enrolled, 82 completed the 9-month follow-up and return-to-sports tests. Of all the patients who were followed-up, 53 passed the RTS tests, and 29 failed due to failures in one or more test items. Fifteen people were randomly selected from the RTS patients and NRTS patients for kinematic evaluation. Among them, 13 males and two females were included in the NRTS group, including 10 right knees and five left knees, and their ages ranged from 22 to 49 years old. A total of 13 males and two females were included in the RTS group, including 11 right knees and four left knees, ranging in age from 21 to 47 years old. There was no significant difference in age, height, weight, BMI, gender composition, or distribution of left and right knee joints between the NRTS group and the RTS group (*p* > 0.05). The clinical characteristics of the two groups of patients are shown in.

### Kinematic evaluation of the knee

**FIGURE 5:**
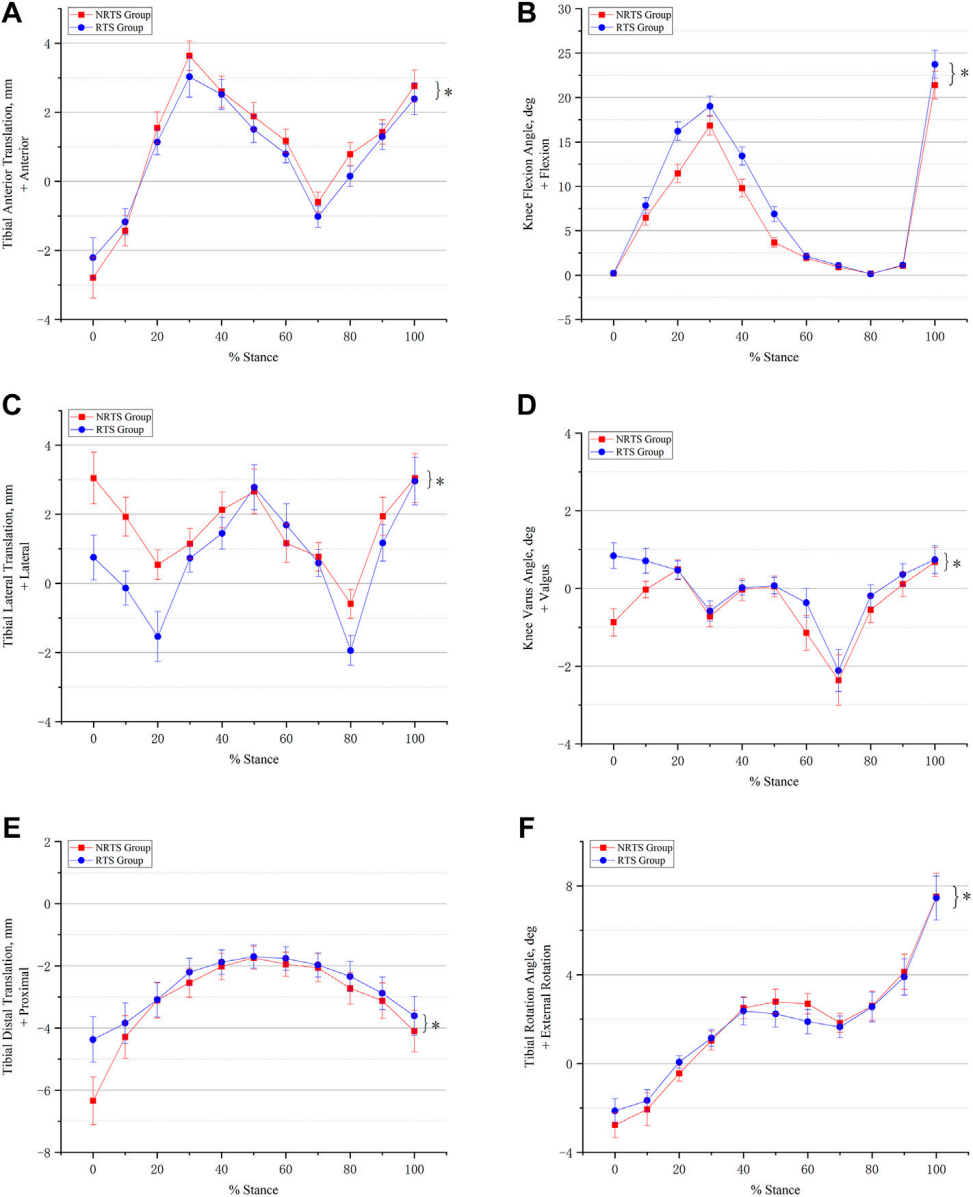
Comparison of six-degree-of-freedom knee kinematics during walking between those who passed the return-to-sports test and those who failed 9 months after ACL reconstruction. **(A)** Anterior-posterior translation. **(B)** Knee flexion and extension. **(C)** Medial-lateral translation. **(D)** Varus-valgus movement. **(E)** Proximal-distal translation. **(F)** External-internal rotation. * Significant difference between groups. NRTS, not return-to-sports; RTS, return-to-sports.

Anterior-posterior translation. The mean anterior tibial translation (ATT) in the NRTS group was greater than that in the RTS group throughout the stance cycle (1.00 ± 0.03 mm vs. 0.76 ± 0.03 mm, *p* = 0.001) (Table 2). Pairwise comparisons showed that the anteroposterior position of the tibia relative to the femur was different between the two groups at 0%, 20%, 50%, 60%, 70%, 80%, and 100% of the support phase cycle. At 0% of the support phase, the ATT in the two groups was the smallest, and the ATT in the NRTS group was smaller than that in the RTS group (−2.79 mm ± 0.15 vs. −2.21 ± 0.15 mm, *p* = 0.01). At 30% of the support phase, the ATT in the two groups was greatest, and the ATT of the NRTS group was greater at this time (3.64 ± 0.42 mm vs. 3.03 ± 0.59 mm, *p* = 0.003) (Figure 5).

Medial-lateral translation. The mean lateral tibial translation of the NRTS group was greater than that of the RTS group during the whole stance cycle (1.61 ± 0.05 mm vs. 0.77 ± 0.05 mm, *p* < 0.001). Pairwise comparisons showed that the medial and lateral positions of the tibia relative to the femur differed between the two groups at 0%, 10%, 20%, 30%, 40%, 60%, 80%, and 90% of the support phase cycle. At 80% of the support phase, the lateral tibial translation in the two groups was smallest and the tibia in the NRTS group was more lateral than that inthe RTS group (−0.59 ± 0.42 mm vs. −1.94 ± 0.43 mm, *p* < 0.001). At 100% of the support phase, the tibia in the two groups was located at the outermost position relative to the femur, and there was no significant difference in the medial and lateral positions of the tibia relative to the femur between the two groups (3.05 ± 0.71 mm vs. 2.96 ± 0.69 mm, *p* = 0.729).

Proximal-distal translation. The tibial movement downwards relative to the femur in the NRTS group during the support phase period was greater than that in the RTS group (−3.09 ± 0.05 mm vs. −2.67 ± 0.05 mm, *p* < 0.001). Pairwise comparisons showed that there was a difference in the up-and-down displacement of the tibia relative to the femur between the two groups at 0%, 80%, and 100% of the support phase cycle. At 0% of the support phase, the downwards displacement of the tibia relative to the femur was the greatest in both groups, at which time the tibia in the NRTS group had a greater downwards displacement than the RTS group (−6.34 ± 0.77 mm vs. −4.37 ± 0.73 mm, *p* < 0.001). At 50% of the support phase, the downwards displacement of the tibia relative to the femur in the two groups was the smallest, and there was no significant difference in the medial and lateral positions of the tibia relative to the femur between the two groups (−1.74 ± 0.37 mm vs. −1.70 ± 0.37 mm, *p* = 0.763).

Flexion-extension. The mean knee flexion angle (KFA) of the NRTS group was lower than that of the RTS group during the stance phase (6.72 ± 0.07° vs. 8.34 ± 0.07°, *p* < 0.001). Pairwise comparisons showed that there were significant differences in KFA between the two groups at 10%, 20%, 30%, 40%, 50%, 70%, and 100% of the stance cycle. At 80% of the stance phase, the KFA of the two groups was the smallest, but there was no significant difference between the two groups (0.13 ± 0.08° vs. 0.18 ± 0.08°, *p* = 0.084). At 100% of the support phase, the KFA of the two groups was the largest, and the KFA of the RTS group was significantly greater than that of the NRTS group (23.74 ± 1.56° vs. 21.39 ± 1.54°, *p* < 0.001).

Varus-valgus. The mean varus angle of the knee in the NRTS group was larger than that in the RTS group in the whole stance phase (−0.40 ± 0.03° vs. −0.01 ± 0.03°, *p* < 0.001). The pairwise comparison results showed that there were significant differences in the valgus and varus angles of the knee between the two groups at 0%, 10%, 60%, 80%, and 90% of the stance cycle. At 70% of the support phase, the knee varus angle of the two groups was the largest, but there was no significant difference in the knee varus angle between the two groups (−2.11 ± 0.54° vs. −2.36 ± 0.65°, *p* = 0.262).

Internal-external rotation. The average external rotation angle (ERA) of the knee in the NRTS group was greater than that in the RTS group during the stance phase cycle (1.80 ± 0.05° vs. 1.77 ± 0.05°, *p* < 0.001). The pairwise comparison results showed that there were significant differences in the internal and external rotation angles of the knee joint between the two groups at 0%, 20%, 50%, and 60% of the stance phase cycle. At 0% of the stance phase, the internal rotation angle (IRA) of the knee joint in both groups was the largest, and the IRA of the NRTS group was significantly larger than that of the RTS group (−2.77 ± 0.57° vs. −2.13 ± 0.55°, *p* = 0.004). At 100% of the support phase, the ERA of the knee was the largest in the two groups, and there was no significant difference in the ERA between the two groups (7.52 ± 1.05° vs. 7.46 ± 0.99°, *p* = 0.871).

## Discussion

The aim of this study was to compare the kinematic differences of the knee joint in the support phase during the level walking process between a return-to-sports (RTS) group and a nonreturn-to-sports (NRTS) group 9 months after ACL reconstruction. This study found that, compared with the RTS group, the NRTS group had significant differences in 6-DOF knee kinematics during walking. First, the mean and maximum anterior tibial displacement in the NRTS group were greater than those in the RTS group during the stance phase (1.00 ± 0.03 mm vs. 0.76 ± 0.03 mm, *p* = 0.001; 3.64 ± 0.42 mm vs. 3.03 ± 0.59 mm, *p* = 0.003). This result suggests that patients who do not return to sports after ACL reconstruction have instability in the anterior-posterior direction of the knee. In fact, the anteroposterior stability of the knee joint is closely related to the function of the ACL (Wang et al., 2021). Li C et al. found that there was more anterior tibial translation in ACL-deficient knees than in healthy control knees during gait (Li et al., 2022). Anterior tibial translation was greatly reduced after ACL reconstruction (Gao and Zheng, 2010). Currently, the relationship between anterior knee stability and return-to-sports after ACL reconstruction is still being explored (Keizer et al., 2021; Kamada et al., 2022). Faleide et al. measured the anterior tibial translation of the knee joint with KT-1000 and found that it was one of the RTS factors (Faleide et al., 2021). Some studies also found that the anterior stability of the knee joint after ACL reconstruction is closely related to the functional performance (Andrä et al., 2021). Therefore, the restoration of anterior stability of the knee joint after ACL reconstruction is of great significance for the recovery of knee joint function and even the return to sports.

In addition, NRTS patients have significant lateral and rotational instability with larger mean lateral tibial translation (1.61 ± 0.05 mm vs. 0.77 ± 0.05 mm, *p* < 0.001) and average external rotation angles (1.80 ± 0.05° vs. 1.77 ± 0.05°, *p* < 0.001). These knee instabilities manifestations have been widely reported in activities such as walking and single-leg hopping after ACL reconstruction (Hofbauer et al., 2014; Shabani et al., 2015). This study found that lateral and rotational instability of the knee is more severe in patients who did not pass RTS tests after ACL reconstruction. In addition, this study found an increased tibial-femoral distance in NRTS patients, especially in the early and late stages of the support phase (−6.34 ± 0.77 mm vs. −4.37 ± 0.73 mm, *p* < 0.001; -4.10 ± 0.67 mm vs. −3.61 ± 0.62 mm, *p* = 0.046). According to previous studies (Liu et al., 2018), an increased tibial-femoral distance can reduce the tibiofemoral contact area and ultimately influence knee rotation and lateral instability. Therefore, the lateral and rotational instability of the knee joint after ACL reconstruction is an important problem. This study found that there was an increased tibiofemoral distance and lateral and rotational instability of the knee in NRTS patients, which needs further investigation.

During the stance phase, there was a decrease in knee flexion in the NRTS group compared with the RTS group (6.72 ± 0.07° vs. 8.34 ± 0.07°, *p* < 0.001). Reduction of the knee flexion angle after ACL reconstruction is a common problem and has been reported in many studies (Goetschius et al., 2018). This study found that the reduction in flexion angle during walking was more obvious in NRTS patients. There has been some progress in the study of decreased knee flexion angles after ACL reconstruction. Blackburn et al. (Blackburn et al., 2019) found that abnormal coactivation of the quadriceps and hamstrings was associated with a decrease in knee flexion during the stance phase. The abnormal joint action of the quadriceps femoris and hamstrings during the support phase makes the knee joint more “stiff”, which is manifested as a decrease in the flexion angle at each moment and a decrease in the overall range of flexion and extension. In addition, it has been reported that a decrease in the knee flexion angle during the support phase is also related to the occurrence and development of osteoarthritis (Pamukoff et al., 2018). To clearly determine the relationship between a reduced knee flexion angle and RTS, more research is needed in the future.

Finally, this study still has the following shortcomings. Due to the workload, it did not accurately calculate the relative position of the tibiofemoral joint in each frame of images during the stance phase, because this takes a lot of time. Therefore, this study refers to the practice of similar previous investigations; that is, the images at every 10% point in the support phase cycle are selected for calculation (Zhang et al., 2021). Since walking is a continuous process, these time points can already well describe the kinematic changes in the whole process. Therefore, we believe that this will not have a significant impact on the conclusions of the experiment, as has been confirmed in the previous literature.

## Conclusion

There are still 35.6% of patients who fail the return-to-sport tests and are not allowed to return to sports 9 months after ACL reconstruction. Compared with those who pass the RTS tests, patients who do not pass show significant anterior, lateral, and rotational instability; knee laxity; and reduced flexion angle of the knee during the support phase during walking. Therefore, the current RTS tests are good at identifying patients with abnormal knee kinematics. The causes and mechanisms of abnormal kinematic performance in patients who fail RTS tests after ACL reconstruction remain to be further studied.

## Data Availability

The raw data supporting the conclusions of this article will be made available by the authors, without undue reservation.
